# Developing an evaluation strategy in Kashmir: assessing the impact of an arts intervention with school children in an area of conflict

**DOI:** 10.1177/17579139241287020

**Published:** 2024-11-06

**Authors:** NJ Holt, M Buser, E Brännlund, J Mytton, L Leeson, V Sinha, A Roy

**Affiliations:** Associate Professor, School of Social Sciences, University of the West of England (UWE Bristol), Frenchay Campus, Coldharbour Lane, Bristol BS16 1QY, UK; University of the West of England (UWE Bristol), UK; Mid Sweden University, Sweden; University of the West of England (UWE Bristol), UK; Middlesex University, UK; Building on Art, India; Katkatha Puppet Arts Trust, India

## Background to the Project

About one in six children live in areas of conflict globally, with significant impact on mental health, behaviour, and life outcomes.^1^ Research on ways to help prevent and reduce suffering is paramount, yet assessing the impact of interventions on children in conflict contexts is challenging, beset with ethical, cultural, and psychometric difficulties.^2^ This practice report shares and reflects on the strategy developed to evaluate the impact of an arts intervention in the Kashmir Valley. This is a highly militarised area, where children were regularly exposed to violence, protests, and lockdowns, which severely impacted education and family life. In June 2020, conditions were compounded by the restrictions imposed by COVID-19. The arts-based intervention was conducted in one school and ran throughout the academic year (from August 2020). Thirty children (aged 10–15) were referred by the school to participate in a programme that was integrated into the curriculum. It included a range of art activities designed to enable expression and improve wellbeing, led by an artist and art therapist.^1^

## Co-Production of the Evaluation Method

The research aimed to deepen understanding of the benefits and barriers to using art-interventions for children in conflict areas using mixed methods. The project was guided by a realist evaluation strategy. Over two months, team members (school staff, in-country artist/art therapist, UK-based academics from multiple disciplines) met online to develop a programme theory (outlining anticipated changes in child and community wellbeing due to the arts programme) and to co-produce methodology. During this process, it was agreed that a non-stigmatising approach, avoiding language that indicated a focus on mental health, or asking children directly about their own mental health and traumatic experiences, was important, for various reasons: trust; safety; cultural context; and narrative.

**Figure 1:**
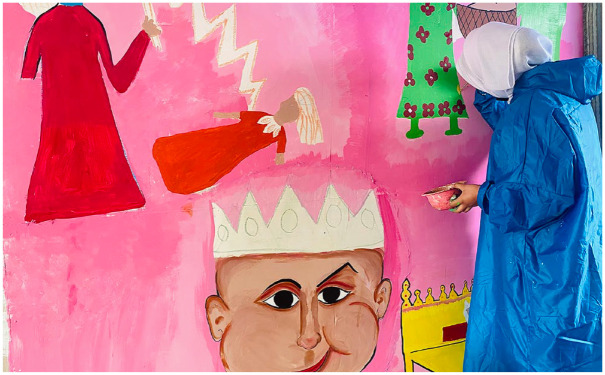
Participant painting a mural during The Art of Healing programme, Kashmir

In terms of trust, the therapeutic alliance between the artists and children was considered; asking direct questions or administering questionnaires about mental health involved methods of disclosure that could negatively impact relationship building and power balance.^3,4^ Especially in the context of trauma, power inequalities introduced into the research process may enhance feelings of a lack of power held at community level, and suggest a hierarchical relationship that is detrimental to trust.^2^ In terms of safety, there was a concern that administering questionnaires on trauma and mental health may increase distress among children, especially if asked for information that is difficult to articulate verbally or forces recall and disclosure of difficult experiences.^5,6^ Distress in response to such methods is predicted by symptoms of mental health and post-traumatic stress,^2^ and while the use of mental health questionnaires has been reported as being acceptable to children,^7^ this is supported by research in Western settings, and does not consider the impact on consequent therapeutic alliance. In terms of cultural context, high levels of stigma towards seeking treatment for mental health have been reported in Kashmir, with fears among adolescents that disclosure could have negative impacts, for example, on future employment.^8^ It was a concern that asking questions about mental health, and framing project involvement in these terms, would be met with resistance by parents and children, potentially decreasing engagement, and consent to participate in the programme. Finally, methods to enable expression of children’s narratives in a meaningful and engaging way were important (without having to speak). Hence, the art programme included (and documented) storytelling, writing, performance, puppetry, and visual art (as illustrated in Figure 1).^3,5^

## Observational, Qualitative, and Arts-Based Methods

It was a challenge to develop mixed methods to assess the wellbeing impact of the intervention unobtrusively. The qualitative evaluation focused on visual and storytelling outputs and end-of-programme interviews with children about their experience of the programme (integrated into its celebration and closure), analysed through narrative and thematic analyses.^1^ To augment this, we included psychometric tools to assess change (Table 1), some of which had previously been used to assess the mental health of children in the context of trauma.^9–11^ We triangulated perspectives, integrating observations of researchers, teachers, and artists, with the children’s narratives.

**Table 1.** Quantitative tools used to assess children’s wellbeing.

Tool	Authors	Domains assessed	Method	Person observing/scoring
Child Behaviour Checklist (CBC)	Achenbach^9^	Anxious/depressed; withdrawn/depressed; somatic complaints; social problems; thought problems; attention problems; rule-breaking behaviour; aggressive behaviour.	Observation of specific recent behaviours in school setting	School teachers
Art Therapy Checklist (ATC)	Save the Children^10^	Body (e.g. motor movements); sensory (e.g. engagement with stimuli); mindfulness; cognitive (e.g. interpretation of story); expressive use of arts; communication; group interaction.	Observation of recent behaviour on the art programme	Artists/art therapist
Art Observation Scale (ArtsObS)	Fancourt and Poon^4^	Anxiety/calmness; sadness/happiness; engaged/non-engaged; high/low interaction with group.	Observation of behaviour during art workshops	Researchers
Human Figure Drawing Test (HFDT)	Koppitz and De Moreau^11^	Indices of emotional distress (e.g. poor integration of parts of the human figure)	Rating of drawings for specific signifiers	Researchers

## Challenges to Consider in Future Research and Evaluation

The interviews and analyses of art proved to be a successful way to gain insight into the mental wellbeing of the children, their experiences of the intervention, and to shed light on their identities and experiences growing up in a conflict area.^1,12^ While quantitative measures provided useful insights into processes of change, several challenges were identified: context, resource, and training. The COVID-19 pandemic required us to move the art activities, initially, online. Interrupted Internet access and cameras sometimes being turned off meant it was not always possible to observe the behaviour of children using the ArtsObS. Observational data is time-consuming to code and requires training, for consistency (reducing subjectivity and bias in coding), and interrater reliability. The CBC is a reliable and well-validated scale as a clinical tool for use in schools, however, it mandates teachers to observe children at each time point, which may be difficult (and required children to have been in a school setting during that time). We further noticed, when coding the HFDT, that cultural context risked inflated emotional distress scores unless the scoring was adapted. For example, marks on faces should be coded as signifiers of distress; however, children sometimes drew masks on faces, which was normative during the COVID-19 pandemic. Finally, the socio-political context, including lockdowns, regular school closures, and a strict political climate, resulted in practical difficulties to execute the project, which influenced children’s involvement, and impacted the analysis of long-term impacts. The successful (short-/mid-term) implementation of the project was achieved thanks to careful negotiation with stakeholders and the arts team’s sustained engagement in the school, as well as attention to trust, safety, cultural context, and narrative. The mixed data worked together to tell a story about the benefits of the arts programme for children, which was facilitated by the unobtrusive evaluation approach. However, our study illustrates that using observational methods effectively requires significant resource and care must be taken over the cultural appropriateness of measures.

## Conclusion

In this study, the voice of children was enabled through visual art and storytelling, while standardised methods to assess clinical symptoms and engagement were collected through observational tools. Due to the complexities of delivering this project in the context of conflict and the COVID-19 pandemic, there were multiple challenges. The arts-based data and interviews enabled the richest interpretation of outcomes, while the quantitative observational approach was useful but more difficult to implement in this context. We hope that sharing our co-produced methodological approach will be useful for other researchers seeking to evaluate the impact of arts-interventions with children in complex and cross-cultural contexts, seeking to create research that feels safe, trusted, culturally appropriate, and rewarding.
